# MicroRNA-34a functions as a tumor suppressor by directly targeting oncogenic PLCE1 in Kazakh esophageal squamous cell carcinoma

**DOI:** 10.18632/oncotarget.21384

**Published:** 2017-09-27

**Authors:** Xiao-Bin Cui, Hao Peng, Ran-Ran Li, Jian-Qin Mu, Lan Yang, Na Li, Chun-Xia Liu, Jian-Ming Hu, Shu-Gang Li, Yutao Wei, Laibo-Yin, Hong Zhou, Feng Li, Yun-Zhao Chen

**Affiliations:** ^1^ Department of Pathology and Key Laboratory for Xinjiang Endemic and Ethnic Diseases, Shihezi University School of Medicine, Shihezi, China; ^2^ Department of Pathology, Beijing Chaoyang Hospital, Capital Medical University, Beijing, China; ^3^ First Department of Internal Medicine, Xinjiang Production and Construction Corp Hospital of Chinese People's Armed Police Force, Urumqi, China; ^4^ Department of Oncology, The First Affiliated Hospital, Shihezi University School of Medicine, Shihezi, China; ^5^ Department of Thoracic and Cardiovascular Surgery, The First Affiliated Hospital, Shihezi University School of Medicine, Shihezi, China; ^6^ Bone Research Program, ANZAC Research Institute, University of Sydney, Sydney, Australia; ^7^ The People's Hospital of Suzhou National Hi-Tech District, Suzhou, China

**Keywords:** esophageal cancer, PLCE1, miR-34a, tumor suppressor, Kazakh

## Abstract

Esophageal squamous cell carcinoma (ESCC) is one of the frequent malignant tumors with poor prognosis worldwide. Identifying the prognostic biomarkers and potential mechanisms of such tumors has attracted increasing interest in esophageal cancer biology. Our previous study showed that phospholipase C elipson 1 (PLCE1) expression is up-regulated and associated with disease progression in esophageal carcinoma. MicroRNAs (miRNAs) play vital roles in regulating its target gene expression. However, studies on miRNA-regulated PLCE1 expression and its cellular function are still very few. We found that miR-34a is significantly expressed lower in ESCC tissues. We further showed that PLCE1 is a direct functional target gene of miR-34a, and the functional roles of miR-34a in ESCC cell lines *in vitro* were also determined through gain- and loss-of-function analyses. Results revealed that miR-34a functions as a tumor suppressor by inhibiting the proliferation, migration, and EMT phenotype, as well as promoting apoptosis of ESCC cell lines. Moreover, PLCE1 is overexpressed in ESCC tumors and promotes tumorigenicity *in vivo* and vitro. PLCE1 expression is negatively correlated with miR-34a profiles in ESCC tissues. Our data suggest that miR-34a exerts its anti-cancer function by suppressing PLCE1. The newly identified miR-34a/PLCE1 axis partially illustrates the molecular mechanism of ESCC metastasis and represents a new candidate therapeutic target for ESCC treatment.

## INTRODUCTION

Esophageal cancer is a frequent malignant tumor that ranks fifth in incidence worldwide and is the fourth leading cause of cancer-related deaths in China [1]. Approximately 70% of esophageal cancers occur in China, where esophageal squamous cell carcinoma (ESCC) is the major type (>90%) [2]. Compared with the national average level (14.95/100,000) and the Han population in Xinjiang (13/100,000), the higher incidence of Kazakh ESCC in Xinjiang has reached 155.9/100,000 [3]. Although surgical techniques, chemotherapy, radiation, and perioperative management have been developed, the mean five-year survival rate of ESCC is only approximately 10%, indicating that there are confined clinical approaches for early diagnosis and treatment of ESCC [4]. Therefore, determining a prognostic biomarker and underlining the molecular mechanism of ESCC are essential to developing a new therapeutic approach to improve patient outcomes.

In recent years, molecular-targeted therapy for various human cancers has been confirmed, and microRNA (miRNA) has been identified as a potential therapeutic target. MiRNAs are a class of non-coding RNA with 17–25 nucleotides that mostly bind to the complementary sequences in 3′ untranslated regions (UTR) of multiple target mRNAs to modulate gene expression [5]. Recent studies have indicated that the dysregulation of miRNAs is involved in a variety of human cancers and mediate in initiation, promotion, progression, and resistance to cancer chemotherapy [6, 7]. To date, many human endogenous miRNAs have been reported in the oncogenesis and progression of ESCC, such as miR-130b [8], miR-93 [9], and miR-183 [10]. MiR-34a is downregulated and correlated with lymph node status and advanced clinical stage in ESCC, and low miR-34a levels in the cancer cells in ESCC are associated with increased colony formation and decreased apoptotic [11–17]. However, the role of miR-34a is complicated and the investigation between miR-34a and ESCC are little. Therefore, miR-34a expression in ESCC is not very well understood, and the potential mechanism of miR-34a in the biological behavior of ESSC remains unclear.

Phospholipase C elipson 1 (PLCE1) is a new subtype of PLC family and encodes a phospholipase C enzyme [18]. PLCE1 can hydrolyze membrane phosphatidyl inositol 4, 5 diphosphate (PIP2) to produce three phosphoinositide (IP3) and diacylglycerol (DAG) to regulate metabolism, cell proliferation, and differentiation [19]. The importance of PLCE1 is demonstrated by its role in several major human malignant tumors, such as, skin [20, 21], bladder [22, 23], colorectal [24–26], head and neck [27, 28] and ESCC cancers [29–31]. PLCE1 acts as a tumor suppressor in colorectal cancer but functions as an oncogene in ESCC and bladder cancers, indicating that the roles of PLCE1 in different tumors remain distinct. Our previous study proved that the heterozygote of PLCE1 rs2274223 is associated with the susceptibility to HPV infection in Kazakh patients with esophageal cancer [32]. We also confirmed that the expression level of PLCE1 protein in ESCC tumors is higher than those in normal tissues in Kazakh ESCC populations [3, 33], results that successfully replicated the results of Wang LD in Han populations [30]. However, the PLCE1 expression of ESCC in different studies has contradicting results; Hu et al. [24] confirmed that the mRNA expression level of PLCE1 in ESCC is lower than that in normal tissues, but the IHC score of ESCC and normal match are not significantly different. Therefore, investigating the underlying mechanisms that result in the aberrant expression of oncogenic PLCE1 in ESCC is very important.

In this study, qRT-PCR was used to demonstrate the frequent down-regulation and significant correlation of miR-34a with the progression of Kazakh ESCC. The overexpression of miR-34a in ESCC cells remarkably suppressed cell growth and migration *in vitro*. Our study likewise first reported PLCE1 as a direct target gene of miR-34a. The tumor-suppressive function of miR-34a is mediated in part by the suppression of PLCE1 expression. The newly identified miR-34a/PLCE1 axis partially illustrates the molecular mechanism of ESCC metastasis and represents a new candidate therapeutic target for ESCC treatment.

## RESULTS

### MiR-34a is aberrantly down-regulated in ESCC tissues and associated with disease progression

**Figure 1:**
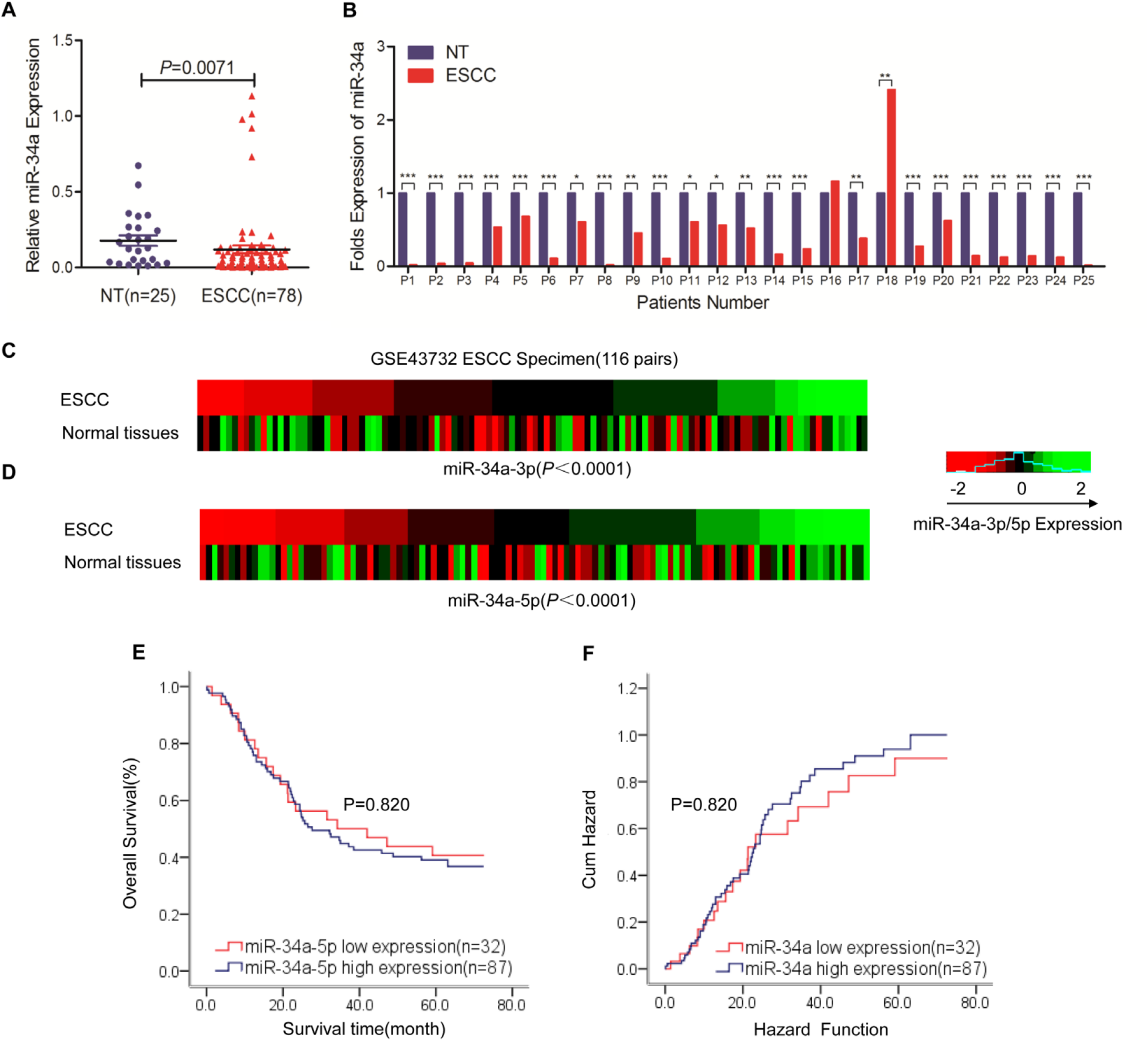
MiR-34a is aberrantly downregulated in ESCC tissues and is correlated with disease progression **(A)** The real-time PCR detection of miR-34a expression in 78 specimens from ESCC cases and 25 specimens from nontumor esophageal cases. miR-34a expression levels were calculated using the miR-34a miR-34a/U6 expression ratio (2 ^− ΔCt^) (^**^P = 0.0071). **(B)** Comparison of miR-34a expression levels between 25 paired ESCC tissues and corresponding nontumor tissues. miR-34a expression was significantly downregulated in 92% (23/25) of all tested esophageal carcinoma tissues compared with miR-34a expression in corresponding nontumor tissues. miR-34a expression was normalized to U6 and calculated by 2^−ΔΔCt^ and then compared with miR-34a expression in normal tissues. **(C-D)** Analysis of microarray data from the Gene Expression Omnibus (GEO) database. The GSE dataset has the accession number GSE43732. Expression of miR-34a-3p and miR-34a-5p in 119 paired ESCC tissues and normal esophageal tissues. **(E-F)** Survival relevance analysis of miR-34a-5p expression in ESCC patients. miR-34a-5p expression was classified as high expression (n = 87) or low expression (n = 32) according to the qRT-PCR results from GEO database. Data were analyzed by a ^−ΔΔCT^ approach and expressed as log2-fold change (^−ΔΔCT^) (mean ± s.d., n = 3, ^*^ *P* < 0.05, ^**^ *P* < 0.01, and ^***^ *P* < 0.001, Student's t-test).

To investigate the roles of miR-34a in ESCC, we first explored the miR-34a expression profiles with quantitative RT-PCR in 78 Kazakh patients with ESCC and 25 non-tumor tissues; 25 pairs were ESCC and their corresponding non-tumorous esophagus. The levels of miR-34a in tumor tissues were found to be significantly lower than those measured in non-tumor tissues (Figure 1A, *P* =0.007). Moreover, comparative analysis indicated that 23 of the 25 (92%) esophageal cancer tissues expressed lower levels of miR-34a compared with the matched non-tumor tissues (Figure 1B). We then summarized miR-34a expression in the large cohort of ESCC patients obtained from the Gene Expression Omnibus (GEO) database of the National Center for Biotechnology Information (NCBI) (accession number: GSE43732). Consistent with our results, both miR-34a-3p and miR-34a-5p levels were significantly decreased in ESCC compared with non-tumor in ESCC patient cohorts (Figure 1C and 1D, P <0.0001; *P* <0.0001). The status of miRNA expression suggested that miR-34a is frequently dysregulated in ESCC.

Clinicopathological analyses of 78 Kazakh ESCC patients showed that a decrease in miR-34a was significantly correlated with lymphatic invasion (*P* =0.014) and tumor-node-metastasis stage *P* =0.018, Table 1). These data indicated that the expression of miR-34a is closely associated with aggressive features and metastatic properties of ESCC. The association between miR-34a expression and patient survival was analyzed to explore the predictive value of miR-34a down-regulation in ESCC. The 119 patients were divided into high (n = 87) and low (n = 32) expression groups according to the signature, and the two groups showed no significantly different survival rates (Figure 1E and 1F, five-year survival: 40.6% vs. 39.1%, *P* =0.820).

**Table 1:** The miR-34a expression and clinic-pathological features in esophageal squamous carcinoma patients

Clinical feature	MiR-34a expression			
	Cases	High (N, %)	Low (N, %)	*P* value
Age				
<65	60	18 (72.0)	42 (79.2)	0.567
≥65	18	7 (28.0)	11 (20.8)	
Sex				
Male	53	15 (60.0)	38 (71.7)	0.436
Female	25	10 (40.0)	15 (28.3)	
Histologic grade				
G1	8	2 (8.0)	6 (11.3)	0.719
G2+G3	70	23 (92.0)	47 (88.7)	
Invasion depth				
T1+T2	59	20 (80.0)	39 (73.6)	0.587
T3+T4	19	5 (20.0)	14 (26.4)	
Lymphatic invasion				
Negative	39	7(28.0)	32 (60.4)	0.014^*^
Positive	39	18 (72.0)	21 (39.6)	
TNM stage				
I/II	55	13 (52.0)	42 (79.2)	0.018^*^
III/IV	23	12 (48.0)	11 (20.8)	

TNM, tumor node metastasis.

χ^2^ test was used to analyze the categorical variables. ^*^P < 0.05.

### MiR-34a down-regulates PLCE1 expression by directly targeting its 3′-UTR

**Figure 2:**
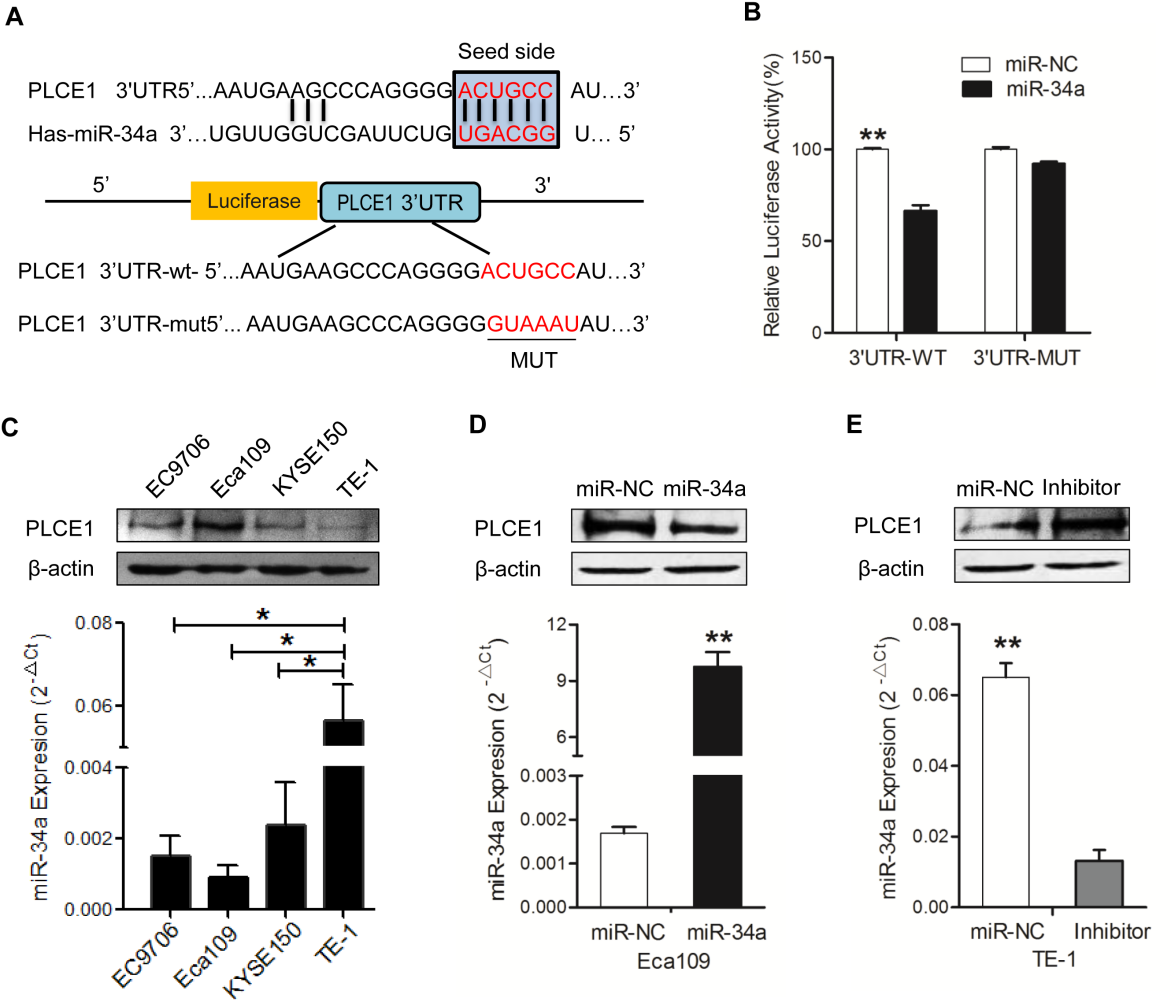
MiR-34a downregulates PLCE1 expression by directly targeting its 3′-UTR **(A)** Prediction of upstream miRNAs targeting PLCE1 by online miRNA target prediction databases (TargetScan, miRanda, and miRDB). **(B)** List of the putative miR-34a binding sequences in the 3′-UTR of PLCE1. Mutations were generated in the PLCE1 3′-UTR sequence in complementary sites for seed regions in miR-34a. A human PLCE1 3′-UTR fragment containing either the wild type or mutant miR-34a binding sequence was cloned downstream of the luciferase reporter gene. **(C)** Analysis of luciferase activity. Eca109 cells were co-transfected with the Renilla luciferase expression construct psiCHECK-2 as the internal control, a firefly luciferase reporter plasmid containing either the wild type or mutant PLCE1 3′-UTR, and either the miR-34a mimic or the negative control (NC). Firefly luciferase activity was normalized to Renilla luciferase activity. Data were from at least three independent experiments. ^*^ *P* < 0.05 and ^**^ *P* < 0.01 compared with controls. **(D-E)** Negative regulation of PLCE1 protein expression by miR-34a. Endogenous PLCE1 protein and miR-34a levels in Eca109 cells transfected with miR-34a mimic or NC, and in TE-1cells transfected with miR-34a inhibitor or NC, were analyzed by Western blot and real-time PCR after 70 h transfection.

This study intended to reveal the functional mechanism of miR-34a through its downstream target genes. Our previous results showed that the increased expression of PLCE1 contributes to the aggressiveness of ESCC, but the molecular mechanism of the aberrant expression remains clear. To investigate if miRNAs are involved in regulating PLCE1 expression in ESCC, three online databases, namely, TargetScan, miRanda, and miRDB, were used to predict the miRNA that can target the PLCE1 of the miRNA; miR-34a gained our attention because it is a well-known anti-oncogene, and the databases predicted miR-34a as a potential miRNA that targets PLCE1. Interestingly, putative binding sites for miR-34a were found in the 3′-UTR of PLCE1 at 218–224 bps, which is highly conserved across species (Figure 2A). To confirm PLCE1 as a direct target of miR-34a, a luciferase reporter assay was performed in Eca-109 cell lines. The relative luciferase activity was significantly reduced in cells co-transfected with miR-34a mimic and 3′-UTR-WT luciferase reporter of-PLCE1. Subsequently, this effect was revived when the 3′-UTR-binding site was mutated, supporting the theory that miRNAs directly regulate PLCE1 by binding to its 3′-UTR (Figure 2B, *P* =0.0019).

Given that PLCE1 was aberrantly overexpressed in ESCC tissues, we hypothesized that the expression of miR-34a can lead to PLCE1 inactivation in ESCC. We detected the endogenous miR-34a and PLCE1 protein levels in various ESCC cell lines. qRT-PCR and Western blot analysis were performed to confirm that miR-34a expression was inversely correlated with PLCE1 expression in four ESCC cell lines (Figure 2C). Compared with Eca-109 cell lines, other cell lines expressed lower PLCE1 protein and higher miR-34a mRNA levels. The Eca-109 cell lines expressed the highest PLCE1 level and lowest miR-34a mRNAs status, whereas TE-1 cells expressed the lowest PLCE1 level and highest miR-34a mRNAs status (*P*=0.0078). The results provided the basis for choosing the two cell lines as vitro cell models to determine the function of miR-34a by overexpression or underexpression. According to the significant inverse correlation between endogenous miR-34a and PLCE1 expressions in ESCC cells, we hypothesized that ectopic miR-34a expression also significantly affects the expression of PLCE1 protein. The successful re-expression and suppression of miR-34a were confirmed by qRT-PCR (Figure 2D, *P* =0.0065; Figure 2E; *P* =0.0038). In the next experiment, the miR-34a mimic significantly suppressed endogenous PLCE1 protein levels in Eca109 cells. We consistently found that the miR-34a inhibitor could increase the expression of endogenous PLCE1 protein expression in TE-1 cells (Figure 2E, *P* =0.0318). Our results indicated that the binding sites may be involved in miR-34a regulation, and that miR-34a directly binds to the 3′-UTR of PLCE1 to regulate its expression.

### MiR-34a functions as a tumor suppressor by inhibiting PLCE1 expression

**Figure 3:**
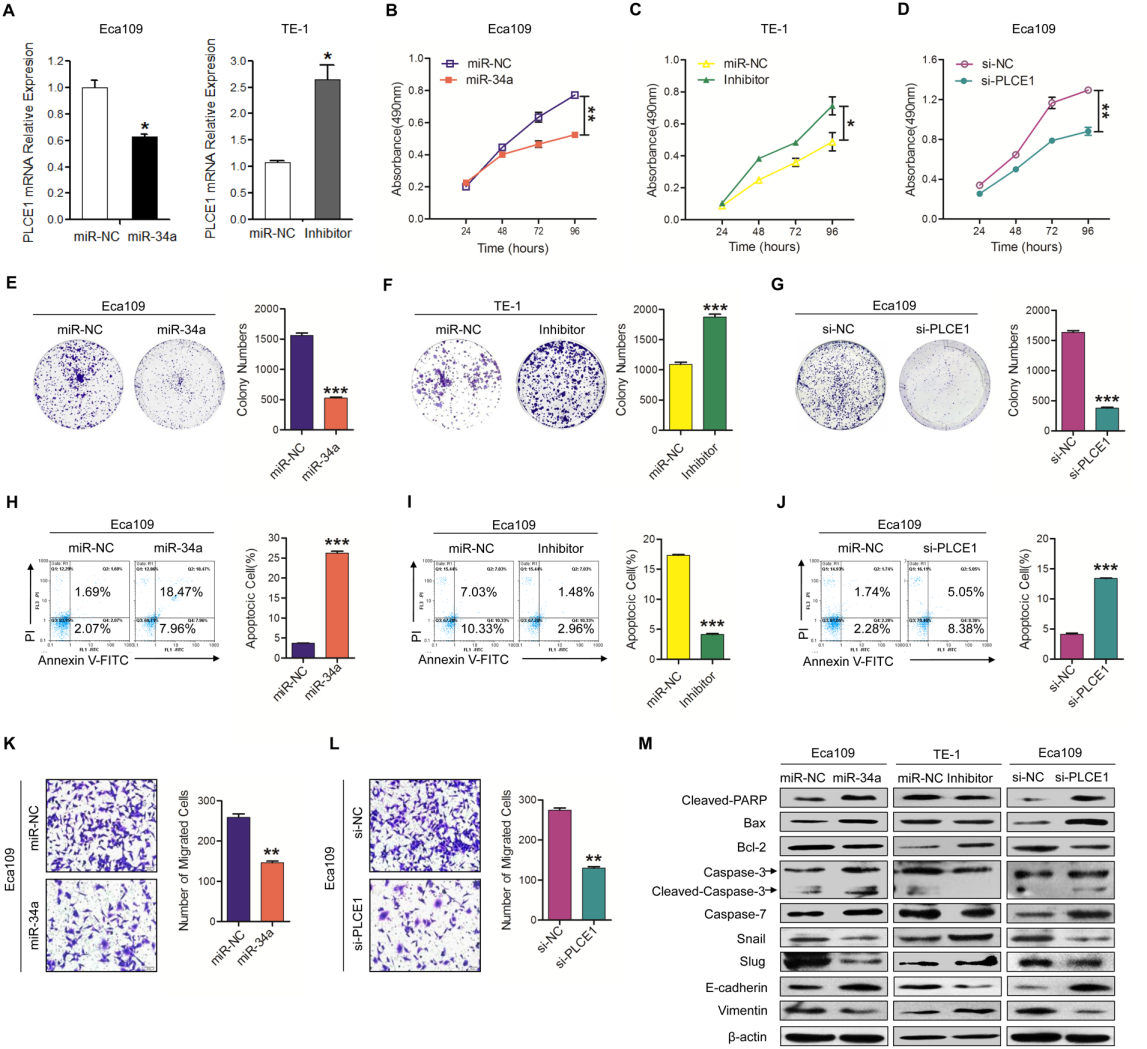
Downregulation of miR-34a-mediated PLCE1 upregulation promotes ESCC aggressiveness *in vitro* **(A)** Endogenous PLCE1 mRNA levels in Eca109 cells transfected with the miR-34a mimic or NC, and in TE-1cells transfected with miR-34a inhibitor or NC, were analyzed by real-time PCR. **(B-D)** Proliferation ability was tested by MTT method in Eca109 cell cultures treated with miR-34a mimic, in TE-1 cell lines treated with miR-34a inhibitor, and in Eca109 treated with PLCE1 siRNA. The experiments were independently repeated three times. **(E-G)** Colony formation assays were performed with cells treated as above. **(H-J)** Evaluation of the effects of miR-34a, miR-34a inhibitor, and si-PLCE1 on cell apoptosis in Eca109 and TE-1 cells using a FACS Calibur flow cytometer. **(K-L)** Transwell assays were performed on Eca109 cell lines treated with miR-34a mimic and PLCE1 siRNA. **(M)** Expression of the apoptosis-related proteins Bax, cleaved PARP, caspase3, cleaved caspase-3, and caspase-7, and of the migration-related proteins snail, slug, E-cadherin, and vimentin were detected by Western blot analysis in the indicated ESCC cell lines. ^*^ *P* < 0.05, ^**^ *P* < 0.01, and ^***^ *P* < 0.001 vs. scramble control (Student's t-test).

Our previous study proved that hyper-methylation-mediated silencing of miR-34a contributes to esophageal carcinoma in the Kazakh population [14]. In numerous studies, miR-34a expression was decreased in many cancer types. To explore the roles of inactivated miR-34a leading to PLCE1 up-regulation in ESCC aggressiveness, Eca109 and TE-1 cells were used in both gain- and loss-of-function analyses. First, qRT-PCR was performed to detect the effects of miR-34a regulation on PLCE1 expression. As shown in Figure 3A, miR-34a can impair the expression of PLCE1. Afterwards, MTT and colony assays were performed to investigate whether miR-34a was responsible for ESCC cell growth and proliferation after regulating PLCE1 expression. MTT assay showed that the miR-34a mimic inhibited cell growth in Eca109 cells (Figure 3B, *P* =0.0031), whereas its inhibitor increased cell growth in TE-1 cells (Figure 3C, *P* =0.0308). Consistent results appeared in colony formation assays (Figure 3E and 3F, P=0.0002; F, *P* =0.0005).

Escaping apoptosis is another superior characteristic of tumor cells for limitless growth. Therefore, flow cytometry analysis was used to detect the effects of miR-34a on ESCC cell apoptosis. The analysis showed that Eca109 cell lines transfected with miR-34a mimic exhibited significantly increased cell apoptosis compared with the controls (Figure 3H, *P* =0.0003). TE-1 cells transfected with miR-34a inhibitor showed opposite alterations (Figure 3I, *P* =0.0003). Western blot analysis exhibited that Cleaved-PARP, Bax, caspase-3, and caspase-7 expression were increased in Eca109 cell lines transfected with miR-34a mimic, whereas inhibited miR-34a decreased their expression. On the other hand, Bcl-2 expression level was decreased in miR-34a mimic-treated Eca109 cells and increased in TE-1 cells treated with miR-34a inhibitor (Figure 3M).

We further investigated whether miR-34a is also involved in the metastatic growth of ESCC. Cell migration assays displayed that miR-34a mimic inhibited cell migration in Eca 109 cells (Figure 3K, *P* =0.003). However, after three repeated experiments, TE-1 cell lines transfected with miR-34a inhibitor and miR-NC failed to penetrate the film and were stuck in the gap of the transwell chamber, making it impossible to count statistical results. Western blot showed that the invasive protein molecules, such as snail and slug, were decreased in Eca109 cells transfected with miR-34a mimic, but increased in TE-1 cells transfected with miR-34a inhibitor (Figure 3M). Moreover, the MET-associated proteins, such as Vimentin and E-cadherin, were also investigated in ESCC cell lines. E-cadherin expression level was increased in miR-34a mimic-treated Eca109 cells and decreased in TE-1 cells treated with miR-34a inhibitor. On the other hand, Vimentin expression level was decreased in miR-34a mimic-treated Eca109 cells and increased in TE-1 cells treated with miR-34a inhibitor (Figure 3M).

Notably, the knockdown of endogenous PLCE1 expression by siRNA significantly suppressed cell growth (Figure 3D, *P* =0.006) and colony formation (Figure 3G, *P* <0.001), but enhanced apoptosis (Figure 3J, *P* =0.0003) and migration (Figure 3L, *P* =0.004) in Eca109 cells; this phenotype was similar to that induced by the overexpression of the miR-34a mimic. We found that miR-34a overexpression or PLCE1 knockdown resulted in the same down-regulation of Bcl-2 and up-regulation of cleaved PARP and Bax (Figure 3M). The results confirmed the effect of miR-34a and supported the major involvement of PLCE1.

### Knockdown of PLCE1 by siRNA can potentiate the antitumor effects of miR-34a

We demonstrated that miR-34a regulates PLCE1 expression in ESCC cells lines. We also detected whether *in vitro* phenotypes associated with miR-34a over-expression could be potentiated by silencing PLCE1 with siRNA. Eca109 cells were treated with miR-34a mimic and PLCE1 siRNA and co-transfected with miR-34a mimic and PLCE1 siRNA. The cells were then tested for their effects on proliferation, migration, apoptosis, and the expression of endogenous proteins.

**Figure 4:**
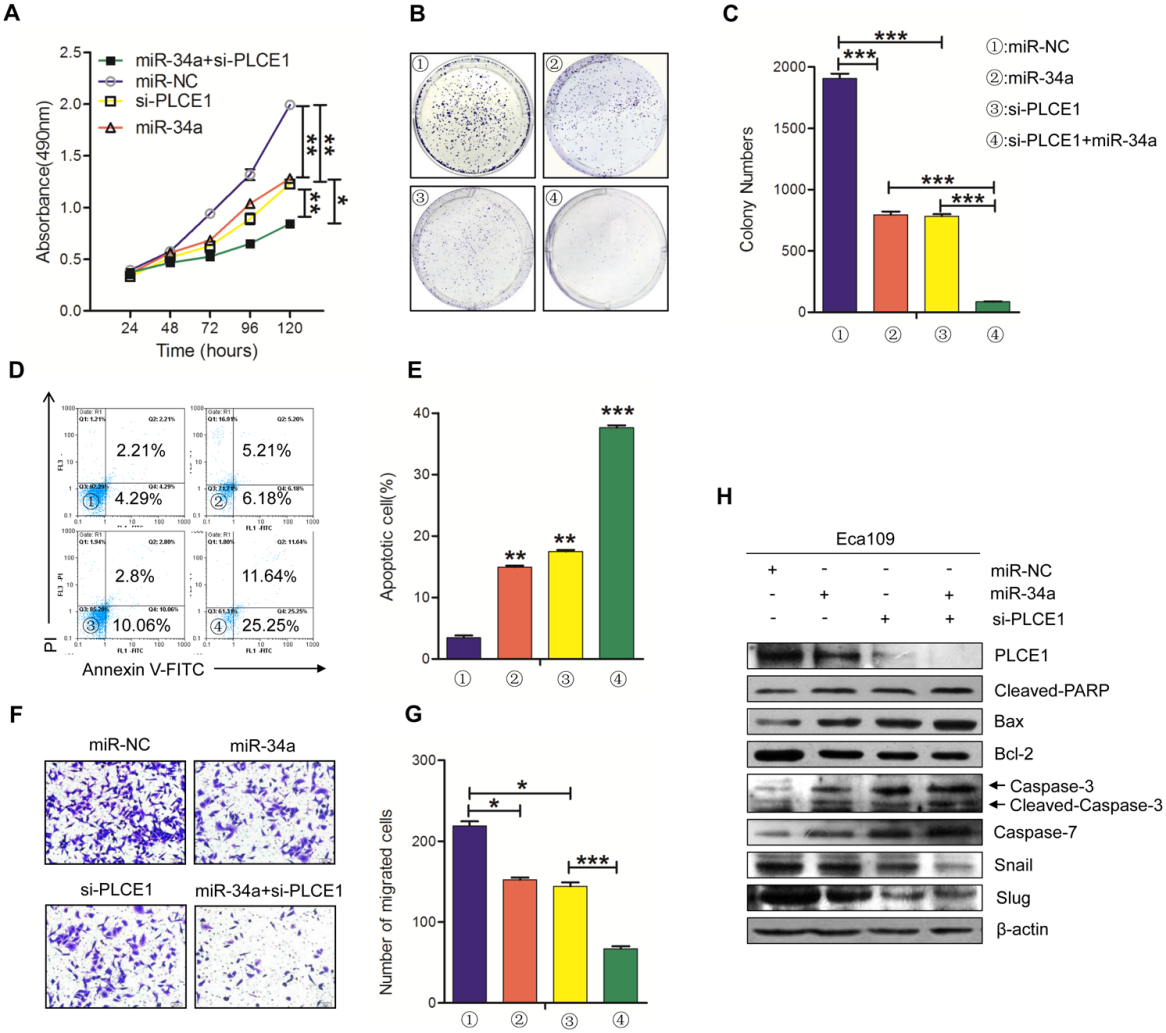
The knockdown of PLCE1 by siRNA promotes the antitumor effects of miR-34a **(A)** Cell proliferation was measured by MTT at 24, 48, 72, 96, and 120 h in Eca109 cells treated with miR-34a mimic, PLCE1 and siRNA co-transfection, or NC. **(B, C)** Representative statistics of colony formation in cell lines with above treatment. Original magnification is 200×. **(D, E)** Cell apoptosis detected by labeled flow cytometry in Eca109 cells at 48 h after transfection. Apoptotic evaluation was performed by calculating the apoptotic percentage of cells. **(F, G)** Ectopic expression of miR-34a promoted the inhibition of cell migration in PLCE1-siRNA- treated cells. Transwell assays were performed 48h after transfection. The representative numbers of cells across a Matrigel membrane with 8 mm pores. (^**^P < 0.01). **(H)** The expression of PLCE1, apoptosis-related proteins, and EMT-related marker proteins in Eca109 cell lines transfected with both miR-34a mimic and PLCE1 siRNA.^*^ *P* < 0.05, ^**^ *P* < 0.01, and ^***^ *P* < 0.001 vs. scramble control (Student's t-test). All results were reproduced by three independent experiments.

First, MTT analysis and colony formation assays were performed to detect cell proliferation. As shown in Figure 4A, the proliferation ability of the Eca109 cells was decreased after transfection with miR-34a mimic (*P* =0.0031) and PLCE1 siRNA (p =0.0046), but a significant decrease was observed in co-transfected cells compared with the two single treatments (*P* =0.0071, *P* =0.0025). The same results in the formation of cloning experiments were discovered (Figure 4B and 4C). The Eca109 cells treated with miR-34a mimic and si-PLCE1 showed decreased colony masses than the controls (*P* =0.0009, *P* =0.0001), and the co-transfected group showed less masses than the two groups (*P* =0.0005, *P* =0.00012). Subsequently, we used flow cytometry analysis and transwell assays to detect their common effects on apoptosis and migration. The knockdown of endogenous PLCE1 expression by siRNA significantly increased cell apoptosis and suppressed migration in Eca109 cells more than the controls (Figure 4D-4E, P =0.0024; Figure 4F-4G, P =0.00128); this phenomenon was similar to that induced by the overexpression of the miR-34a mimic (Figure 4D-4E, P =0.0017; Figure 4F-4G, P =0.0179). The co-current processing group had more significant effects on cells apoptosis and migration than the controls (Figure 4D-4E, P =0. 0003; Figure 4F-4G, P =0.0026). Western blot analysis results showed that similar reductions in PLCE1 and pro-apoptotic protein were induced by PLCE1 siRNA and miR-34a mimic in Eca109 cells, whereas these were increased in anti-apoptotic protein Bcl-2 (Figure 4H). These results suggested that the knockdown of PLCE1 can strengthen the antitumor effects of miR-34a. MiR-34a suppressed PLCE1 expression by binding to the 3′-UTR of the PLCE1, and miR-34a can play a partially antitumor function by negatively controlling PLCE1.

### PLCE1 promotes tumorigenicity *in vivo* and inverse correlation between miR-34a and PLCE1 expression in ESCC tissues

**Figure 5:**
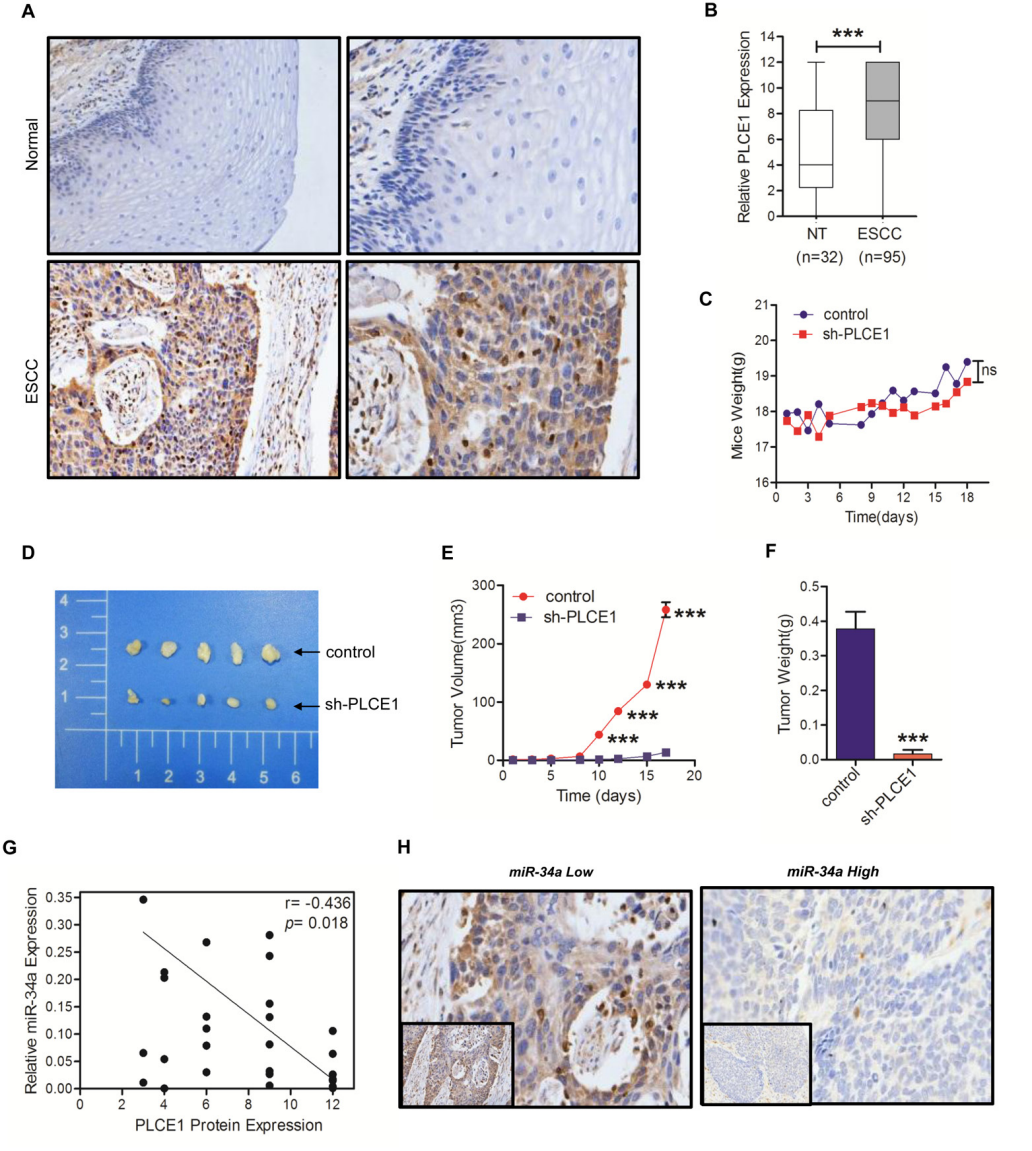
PLCE1 is aberrantly upregulated in ESCC and promotes tumorigenicity *in vivo* **(A)** Representative PLCE1 immunostaining in 98 samples of ESCC tissues and 32 samples of normal tissues. Original magnification is 200×. **(B)** Boxplot analysis of PLCE1 immunohistochemical scores in normal human esophageal squamous tissues and ESCC tissues (Student's t-test). **(C)** Mice transplanted with Eca109 shPLCE1 stable cells (n = 5) or Eca109/vector control cells (n = 5). The weight of mice were recorded at indicated times. **(D)** Image of representative tumors from control or shPLCE1-stable Eca109 xenografts obtained at the end point. **(E)** Xenograft tumor growth was monitored and showed as mean ± s.d (Student's t-test). **(F)** Tumor weights of mice harvested at 18 d after inoculation with Eca109/shPLCE1 cell lines (mean ± s.d., Student's t-test) compared with Eca109/vector cell lines. **(G)** An inverse relationship between miR-34a expression and PLCE1 protein level in ESCC tissues was established by Spearman correlation (r = -04685 with a significant *P* = 0.0189). **(H)** Immunohistochemical staining showing the differential expression of PLCE1 in ESCC with low or high miR-34a expression. ^*^ *P* < 0.05, ^**^ *P* < 0.01, and ^***^ *P* < 0.001.

To validate the previously observed pattern of increased expression level of PLCE1 in ESCC, immunohistochemistry analysis was performed with a tissue microarray containing 100 cases of paraffin-embedded ESCC tissues and 100 cases of paraffin-embedded adjacent nontumourous esophageal tissues. The results revealed that PLCE1 expression was mainly present in the membrane and cytoplasm of Eca cells (Figure 5A). As shown in Figure 5A and 5B, PLCE1 expression in ESCC patients was aberrantly higher than that observed in nontumorous tissues (*P* <0.0001). A high expression (>4 point) of PLCE1 presented in 72% (72/100) of the ESCC tissues, whereas a low expression of PLCE1 presented in 84% (84/100) of the non-tumor tissues. To further determine the role of PLCE1 in esophageal squamous cancer progression, Eca109 cells lines stably lower expressing PLCE1 or the control vector were generated and then orthotopically injected into the dorsal flanks of BALB/c nude mice. All nude mice developed detectable tumors on the same day. As shown in Figure 5C, the body weights of the mice did not differ (*P* =0.0524). After 18 days of inoculation, we found that the tumors formed by Eca109 cells that expressed lower PLCE1 were larger in both size and weight than the control tumors (Figure 5D and 5E, P <0.0001; Figure 5F, *P* =0.0024). These data strongly support that PLCE1 promotes tumorigenicity *in vitro* and *in vivo*.

MiRNAs have opposite expression patterns compared to their targets. To further investigate if miRNA-34a is involved in regulating PLCE1 expression in ESCC, real-time PCR was used to examine the expression levels of miR-34a in 25 paired human ESCC and adjacent normal samples that were subjected to PLCE1 immunohistochemical staining. Among the 25 cases of ESCC tissues, the PLCE1 levels in ESCC patients were also found to be inversely correlated with miR-34a expression in tumor tissues (Figure 5G, r = -0.4658, *P* =0.0189). Similar results can be viewed in the immunohistochemistry images (Figure 5H). These data further supported an oncogenic function of PLCE1 in ESCC. MiR-34a functions as a tumor suppressor in Kazakh populations by inhibiting PLCE1 activity.

## DISCUSSION

Esophageal cancer is one of the most common tumors, and its occurrence and development are complex and continuous processes with multiple factors and steps. Carcinogenic factors can trigger related gene mutations, resulting in abnormal gene expressions that ultimately affect the body tumor, as well as induce tumor growth, invasion, metastasis, and a series of complex processes. Extensive research on the specific mechanism of these processes, with all the protection and regulating mechanism in the process of inhibiting the disorders, is being carried out [34]. The current study confirmed that the aberrant expression of miR-34a is associated with lymph node metastasis and advanced stage in ESCC. Importantly, its target gene, PLCE1, was identified as capable of providing a wide perspective underlying the molecular mechanism of ESCC development, which would be helpful in developing a new therapy approach for ESCC.

Abnormal expressions of miRNAs are involved in various cancer types. Thus far, dysregulated miRNA expression has been implicated in affecting the malignant biological functions of esophageal cancer [35]. MiR-34a is located in chromosome 1p36, which belongs to the tumor suppressor miR-34 cluster [36]. Some studies have reported that miR-34a lacks expression in human esophageal cancer, pancreatic cancer, colon cancer, and neuroblastoma [11, 37–39], and that its low expression promotes tumor formation. We proved the relevance of miR-34a in clinical malignancies, that is, the low expression of miR-34a is significantly associated with advanced stages and lymph node metastasis in Kazakh ESCC patients. Dysregulation of miRNAs can be caused by aberrant DNA methylation, which has been observed to benefit tumorigenesis. Our previous findings revealed that methylation causes a decrease in the expression of miR-34a, and hypermethylation of certain miR-34a sites is related to esophageal advanced cancer stage and lymph node metastasis [14]. Our results are consistent with other research results; for example, Lin et al. and Nie et al. found that miR-34a expression levels in ESCC tissues are significantly lower than those of paired cancer adjacent normal tissues [11, 12]. In addition, we confirmed the role of miR-34a in association with the lymph metastasis and TNM stage in ESCC patients. Consistent with our results that miR-34a down-regulation correlates with the aggressive progression of ESCC, Lin et al. [11] and Hu et al. [15] found that ESCC patients with low tumor tissue expression levels of miR-34a had significantly poor prognoses. We analyzed the data from the GEO database and found no significance between miR-34a expression and patient survival. On the contrary, Lin et al. revealed that low miR-34a profile had a significant effect on the overall survival of patients with ESCC. Therefore, in all future studies, a larger sample size will be used to confirm the correlation of miR-34a levels and patient survival. A multicenter validation of the ESCC samples can be undertaken as well. These findings fully illustrate that miR-34a is a tumor suppressor that influences the progression of ESCC, though the specific mechanisms still need to be studied.

The biological function of miR-34a in ESCC is still poorly understood. Therefore, we verified the effects of miR-34a on cell proliferation, apoptosis, and migration in ESCC cells. Our results showed that miR-34a overexpression significantly suppressed cell proliferation and growth, promoted apoptosis, and inhibited cell migration in ESCC cells. We also showed that miR-34a inhibitor promoted proliferation and migration, but inhibited the apoptosis of ESCC cells. Nie et al. confirmed that miR-34a could inhibit the invasion and migration in ESCC cell lines [12]. Li J et al. also confirmed that miR-34a functions as a tumor suppressor in ESCC cell lines [16], which is consistent with our results. These findings indicate that the down-regulation of miR-34a has an important role in the process of ESCC metastasis and growth, and miR-34a may be a novel potential therapeutic target for ESCC treatment.

MiR-34a exerts its tumor-suppressor function through several targets in certain cancer types [40–42]. To determine the mechanism by which miR-34a regulates ESCC growth and migration, its target genes were measured using publicly available online algorithms (i.e., TargetScan, miRanda, and PITA), and these predictions were validated by luciferase assay. In the present study, miR-34a was identified as a negative regulator of the proliferation, apoptosis, and migration of Eca cell lines by targeting PLCE1 expression (see Figure 2, 3). MiR-34a expression has a negative correlation with PLCE1 protein in Kazakh ESCC tissues. We co-transfected PLCE1 siRNA and miR-34a mimic into the Eca109 cells lines and revealed that elevated PLCE1 expression was more attenuated than the group treated with PLCE1 siRNA or miR-34a mimic only. The results indicated that miR-34a may function as a tumor suppressor gene by at least suppressing PLCE1 expression partially in ESCC. This study uncovered a new mechanism of PLCE1-aberrant expression. In pancreatic cancer, miR-34a inhibits Notch1-induced cell cycle arrest and decreases cell invasion [43]. MiR-34a targets the YY1 in the ESCC. Ma W et al. demonstrated that miR-34a inhibits the proliferative potential of breast cancer stemness *in vitro* and *in vivo* by down-regulating SIRT1 [44]. The results suggested that miR-34a can multitask by regulating different downstream genes in different cancer conditions, and it functions as a metastasis suppressor in ESCC. Furthermore, our previous study showed that PLCE1 inhibits apoptosis by inhibiting the expression of P53, which is consistent with the study of Yun Li et al [3, 45]. Moreover, p53 plays a vital role in activating miR-34a by banding to its promoter [46]. Therefore, PLCE1, whether or not it decreases mir-34a expression by inhibiting the expression of P53, ultimately changes the biological behavior of esophageal cancer cells, though this finding requires further study. Therefore, our research revealed a potential mechanism that may be involved in the abnormal process of miR-34a expression.

Li dong Wang et al. [29–31] and our research group [47] carried out GWAS technology screening of PLCE1 to check it there are susceptibility genes for Chinese Han and Kazak esophageal cancer populations, specifically, if it has a significant relevance with Kazakh ESCC. However, the expression of PLCE1 protein in ESCC was rare. Consistent with the study performed on Han ESCC population by Wang et al., our present study proved that PLCE1 was overexpressed in Kazakh ESCC compared with normal tissues. Our previous study found that ESCC was relatively significantly up-regulated because of metastasis and poor differentiation by interacting with the NF-KB signal pathway [48]. Li Yun et al. reported that the expression of PLCE1 protein in esophageal cancer cells was also up-regulated [45]. However, Hu et al. stated that PLCE1 mRNA was expressed lower in ESCC tissues compared with normal tissues. Moreover, the IHC scores in ESCC and normal tissues were not significantly different from the Han population [24]. Hence, the carcinogenic potential and possible mechanism of PLCE1 dysregulation has not been fully elucidated. Recent studies suggested that PLCE1 expression is regulated by miRNAs in esophageal cancer, and miRNAs regulating PLCE1 are involved in numerous cellular biological behaviors, including cell proliferation, apoptosis, and migration. For instance, our previous study confirmed that miR-145 acts as a tumor suppressor by inhibiting PLCE1 in ESCC [3]. Na Han et al. also demonstrated that miR-328 restrains the survival of EC cells by directly inhibiting PLCE1 expression [35]. Chen G et al. found that PLCE1 is a direct target of miR-1976 and can reverse the tumor suppressor function of miR-1976 by promoting the growth and metastasis of NSCLC cells [49]. However, the studies on miRNAs selectively regulating PLCE1 are few.

Despite extensive studies for several years, the role of PLCE1 in different human tumors remains controversial. Although PLCE1 expression has been found to promote tumor formation in a mouse model of skin cancer, PLCE1^−/−^ mice have exhibited decreased susceptibility to tumor development [21]. In a study by Oka, PLCE^−/−^ mice showed reduced cell death secondary to UVB irradiation compared with PLCE^+/−^ and “PLCE^+/−^ mice [20]. Reduced levels of PLCE1 proteins are present in colon cancer [25, 26, 50, 51]. However, PLCE1 overexpression is found in bladder [23], head and neck cancers [27, 28] as well as ESCC [3, 47], thus indicating the role of PLCE1 in tumorigenesis. The present study proved that PLCE1 functions as an oncogene that can promote tumor growth in *in vivo* experiments. We also used siRNA to silence the PLCE1 gene in ESCC cells, and found that silencing PLCE1 significantly inhibited cell proliferation and migration but increased cell apoptosis rate, a result that is similar to our previous studies. More recently, many studies have suggested that genetic variants in PLCE1 may serve as candidate markers for cancers and influence cancer risk [24, 32, 52]. Our previous study also revealed that single nucleotide polymorphisms (SNPs) are significantly associated with overall risk and various clinicopathological variables in Kazakh ESCC by promoting mRNA and protein expression [47]. Moreover, the heterozygote of PLCE1 rs2274223 enhanced susceptibility to HPV infection in Kazakh ESCC patients [32]. After Ou et al. applied the shRNA-silencing PLCE1 gene in bladder cancer cell line T24 in, expressions of MMP and BCL2 decreased and invasion ability of bladder cancer cells was inhibited, which eventually prevented tumor development [23]. In esophageal carcinoma cells, PLCE1 knockdown increased p53 expression and apoptosis via regulating p53 promoter methylation [45]. Li et al. confirmed that the high level of PLCE1 gene expression in mice can promote the formation of vascular epidermal growth factor receptor-A, thereby enhancing tumor angiogenesis and promoting the occurrence and metastasis of colon cancer [53]. These findings indicated that the genotype-phenotype of PLCE1 may affect epidemiologic and etiologic factors involved in ESCC tumorigenesis, which contributes to the risk for esophageal cancer. However, the PLCE1 gene, whether as an oncogene or tumor suppressor gene, and how it affects the development of ESCC, need further study.

In conclusion, our results identified a novel proliferation and metastasis suppressor, miR-34a, which is a negative regulator of PLCE1. Our study suggests that miR-34a and PLCE1 are significant biomarkers for metastasis. The newly identified miR-34a/PLCE1 axis provides new insight into the nosogenesis of ESCC, particularly with respect to proliferation, apoptosis, and migration, as well as represents a potential target for developing therapeutic agents for ESCC treatment.

## MATERIALS AND METHODS

### Tissue samples

A total of 148 cases of paraffin-embedded Kazakh ethnic ESCC tissues and 58 cases of non-tumor esophageal tissues were collected between 2009 and 2011 from the Department of Pathology Shihezi University, Xinjiang Uygur Autonomous Region, China. Among these, 25 pairs of ESCC and corresponding nontumorous esophageal tissues were collected in parallel. No patient had received radiotherapy or chemotherapy before surgery. No restrictions regarding age, sex, or disease stage were set. Clinical data on clinic-pathological variables, such as invasion depth, histologic grade, lymphatic invasion, and TNM stage, were also gathered from the medical records of the patients. The TNM stages were evaluated based on the seventh edition of the Cancer Stage Manual edited by the American Joint Committee on Cancer in 2009. The use of human tissues was approved by Shihezi University and the Institute Research Ethics Committee. Histopathologic slices were diagnosed by two pathologists, and histopathological grades and classification were in accordance with the “WHO Classification Tumors of the Digestive System” edited in 2010.

### Detection of PLCE1 expression by immunohistochemistry using TMAs

We sampled 95 cases of paraffin-embedded Kazakh ethnic ESCC tissues and selected all 32 cases of adjacent normal tissue samples with 1.0 mm diameter tissue cores using a tissue arrayer (ALPHELYS, Plaisir, France). After fixing with 10% formalin in PBS, the 4 μm paraffin-embedded sections were baked at 65°C for 1 h, and then rehydrated using graded alcohols. Each 4 μm tissue section was deparaffinized and rehydrated. The experimental use of antigen EDTA high pressure repair were as follows. The sections were placed in a plastic repair box containing an EDTA buffer in a microwave oven at 130°C for 10 min. The repair box was then removed and allowed to stand in room temperature with 3% H2O2 added, and then incubated for 10 min to block endogenous peroxidase. The diluted PLCE1 antibody (HPA015598; Sigma-Aldrich Co. St. Louis, MO, USA; 1:50 dilutions) was added to the tissue sections, placed in a refrigerator at 4°C, and incubated overnight in a wet box. The sections were washed with PBS and incubated with secondary antibodies at 37°C for 30 min. Next, 3,3-diaminobenzidine was used to visualize PLCE1 antibody binding, and the tissue sections were counterstained with hematoxylin. After counterstaining, the sections were dehydrated and sealed.

### Immunohistochemical score

Brown granules in the cytoplasm were determined to be positive for PLCE1 protein staining. The results were judged separately by two pathologists, and the inter-observer variability was determined to be <3%. Immunohistochemistry score (Immunoreactivity Score, IS) was judged according to the equation stained area × staining intensity. The percentage of positively stained cells was scored as follows: 0 point (<5% positive cells), 1 point (6%–25% positive cells), 2 points (26%–50% positive cells), 3 points (51%–75% positive cells), and 4 points (>75% positive cells). The scoring rules for cytoplasmic staining intensity were as follows: 0 point, no color; 1 point, buff; 2 points, yellow; and 3 points, brown. Hence, the range of IS was from 0 to 12 points.

### Cell culture and transfections

Eca109, KYSE150, EC9706, and TE-1 cells were obtained from the Institute of Biochemistry and Cell Biology of the Chinese Academy of Sciences (Shanghai, China). The cell lines were cultured in an RPMI-1640 medium containing 10% heat-inactivated fetal bovine serum, 100 units of penicillin/ml (Sigma), and 100 mg of streptomycin/ml (Sigma). All the cell lines were incubated in an incubator at 37°C and 5% CO_2_. The miRNA-34a mimics, miRNA 34a inhibitor, PLCE1 siRNA, and relative negative scramble control RNAs were synthesized by Qiagen Company (Hilden, Germany). Eca109 cell lines were transfected with miR-34a mimic, si-PLCE1, and relative negative scramble control RNAs; and TE-1 was transfected with miR-34a inhibitor and negative control plasmid. The target sequences of miRNA-34a mimics, miRNA 34a inhibitor, and PLCE1 siRNA are as follows: has-miR-34a mimic has 5′-UGGCAGUGUCUUAGCUGGU UGU-3′, anti-has-miR-34a inhibitor has 5′-UGGCAGUGUCUUAGCUGGUUGU-3′, PLCE1- siRNA1 has 5′-AGC GUU GGU CCA UGC UUA ATT-3′, and PLCE1-siRNA2 has 5′-GGG UCU UGC CAG UCG ACU ATT-3′. The miR-34a mimic, inhibitor, PLCE1 siRNA, and negative control plasmid were transfected into ESCC cell lines by HiPerFect reagent and prepared for the following experiments.

### RNA isolation and quantitative real-time PCR

Total RNA was isolated from 78 cases of ESCC tissues, 25 cases of non-tumor tissues, and ESCC cell lines transfected with miR-34a mimic, inhibitor, si-PLCE1, and their own blank vector using a miRNA Extraction Kit (Qiagen, Hilden, Germany). Among the tissue samples, there were 25 paired ESCC tissues and their corresponding non-tumor tissues were selected randomly. Reverse transcription for the quantification of miRNAs was conducted via miRNA cDNA Synthesis Kit (Qiagen). The qPCR amplification of miR-34a was executed via SYBR green Premix Ex Taq II (Qiagen) using Step One Plus Real-Time PCR System (Applied Biosystems). The expression level of miRNA was normalized using U6 as an internal control. The experiment was executed in ABI Prism 7500 Sequence Detection System. The reaction was carried out with an ABI Prism 7500 Sequence Detection System (Applied Biosystems, Foster City, CA, USA). The expression level of miR-34a gene in all cases was calculated by 2^−ΔCt^ method, ΔCt = Ct miR-34a - Ct U6. The difference of miR-34a expression in ESCC and corresponding normal tissues was represented by the RQ value, which was 2^−ΔΔCt^ (ΔΔCt = ΔCt ESCC-ΔCt corresponding normal tissues), indicating the significant miR-34a gene expression of ESCC in tissues compared with adjacent normal tissue. Primers were used as follows:

MiR-34a: 5′-CCCAGAACATAGACACGCTGGA-3′

U6: 5′-TGGTGAAGACGCCAGTGGA-3′

### Western blot analysis

Cells were collected and lysed in RIPA lysis buffer (Solarbio). A total of 10 μL of each protein extract were added into 6%–15% SDS-PAGE gels and transferred to PVDF membrane (Immobilon 0.45 μm, Millipore, USA). The resulting blots were immersed in the first antibodies diluted with 5% non-fat milk overnight at 4°C, including PLCE1 (sc-28404, 1:200), Bax (ab32503, 1:1000), cleaved-PARP (ab32064, 1:5000), Snail (13099-1-AP, 1:250), Slug (12129-1-AP, 1:250), caspase-3 (19677-1-AP, 1:250), caspase-7 (BA0688-1, 1:125), Bcl-2 (AB112, 1:1000), and β-actin (Zhongshanjinqiao 140411, 1:1000); and then treated with a secondary antibody for 2 h at room temperature. Finally, the resulting bands were exposed using the standard ECL procedure. Protein was normalized with β-actin.

### Luciferase reporter assay

For the luciferase reporter assay, Eca109 cells were co-transfected with 20 mM miR-34a mimic or the negative control and 200 ng of psiCHECK-2-PLCE1-3′-UTR-WT, psiCHECK-2- PLCE1 -MUT, and 10 ng Renilla luciferase vector via Lipofectamine2000 Reagent (Invitrogen). After 48 h of transfection, cells were collected and analyzed with the Dual-Luciferase Reporter Assay System (Promega, CA, USA). Luciferase activity was detected by the GloMax fluorescence reader (Promega). The pRL-CMV sea renal fluorescent was utilized as a control for contrast correction in transfection efficiency. The experiments were repeated thrice.

### 2.8. 3-(4, 5-Dimethyl-2-thiazolyl)-2, 5-diphenyl-2H-tetrazolium bromide (MTT) assay

A total of 4000 cells were inoculated into the 96-hole plate, and each group was treated with 3 holes. The cells were incubated in the incubator at 37°C and 5% CO2. After transfection at 24, 48, 72, and 96 h, each hole (including zero adjustment holes) joined MTT 20 μl to remove a 96-hole plate for testing. After 4 h, 150 μL DMSO was added into each hole. We observed the 490 nm OD values of cells with an enzyme labeled detector, repeated this step thrice, and took the average value and actual value of the cell in each hole minus the zero pore. The cell growth curve was made according to the recorded value.

### Colony formation assay

A total of 3000 cells were seeded in 6-well plates for transfection. After 15 days of culture, they were gently rinsed with PBS and fixed for 30 min with 4% paraformaldehyde, and then stained with 0.1% crystal violet for 20 min for image acquisition. Gel-Pro analyzer 4 was used to record the number of cloned cell mass.

### Cell apoptosis assay

After 48 h of transfection, cells were washed thrice with PBS and digested with 200 μL trypsin without EDTA for 1 min in 24-well plates. The cells were collected, centrifuged, and resuspended with 1 × Binding Buffer in a 500 μl/tube. Except for the zero hole, each tube was added with 5 μl FITC and 10 μl PI and then kept in the dark for 5 min. Afterward, 2 ml PBS was added into each flow tube, and the treated cells were assessed via flow cytometry.

### Transwell assay

We took out the cells transfected for 48 h after trypsin digestion and dispersion and then counted. Next, 600 μL of complete medium containing 20% FBS was added into the lower chamber, while 200 μl serum-free cell suspension containing 5 × 10^4^ cells was added into the upper chamber. These were then placed in an incubator at 37°C with 5% CO_2_. After 24 h, the chamber was taken out, rinsed with PBS twice, and then fixed in 4% paraformaldehyde for 20 min at 4°C. Cells were stained with 600 μl 0.1% crystal violet per hole for 15 min and then washed. Next, the inside of the cell membrane was gently wiped with a cotton swab. Under the 5 field of microscope of 200×, the cell count was taken as the average of the number of cells within the hole.

### Tumorigenicity assays in nude mice

Animal studies were conducted according to the principles approved by the University of Minnesota Institutional Animal Care and Use Committee by our laboratory and Tianjin Saierbio Company. Female BALB/C nude mice (aged 4–5 weeks) were divided randomly into experimental and control groups (n = 10 per group). The experimental group was injected with 2 × 10^6^ Eca109 cells that had been stably transfected with PLCE1 siRNA lentivirus; and the control group received 2 × 10^6^ Eca109 GEF control cells at the left axillary subcutaneous tissue. The tumor volume and weight of the nude mice were observed upon tumor formation, and the growth of the tumor was observed every three days. The tumor volumes were calculated via the formula length × width × height. After 35 days of inoculation, nude mice were anesthetized with 1% pentobarbital and observed via IVIS imaging system (Caliper Life Sciences, USA). The mice were then sacrificed, and the stripping surgery of the tumor was weighed and photographed. The tumor volume was measured before and after treatment, and the growth curve of tumor in the nude mice was drawn. Each nude mouse tumor tissue was fixed in 4% formalin.

### Statistical analysis

The SPSS 13.0 software was used for statistical analysis. The miR-34a expression was expressed as mean ± marked difference (X ± S). The paired sample t test was used on PLCE1 protein expression quantity expressed in the median and quartiles via Wilcoxon signed rank and inspection. The χ^2^ test was used to analyze the relationship between the levels of miR-34a expression and clinicopathologic characteristics. Spearman's correlation analysis was used to determine the correlation between PLCE1 and miR-34a expression. If *P* <0.05, then the difference has statistical significance, with “^*^” mark; if *P* <0.01, with “^*^” mark; if *P* <0.001, with “^**^”. The data chart was produced by using Prism Graphpad 5 software.
